# The influence of longitudinal mentoring on medical student selection of primary care residencies

**DOI:** 10.1186/1472-6920-11-27

**Published:** 2011-06-02

**Authors:** Diane Indyk, Darwin Deen, Alice Fornari, Maria T Santos, Wei-Hsin Lu, Lisa Rucker

**Affiliations:** 1 Department of Pediatrics, Jacobi Medical Center of Einstein College of Medicine, Bronx, NY, USA; 2 Department of Medicine, Sophie Davis School of Biomedical Education, City College of New York, NY, USA; 3 Office of Faculty Development, Hofstra University School of Medicine in partnership with North Shore-Long Island Jewish Healthy, Long Island, NY, USA; 4 Department of Family Medicine, Einstein College of Medicine, Bronx, NY, USA; 5 Office of the Dean, Stony Brook University School of Medicine, Long Island, NY, USA; 6 Department of Medicine, Jacobi Medical Center of Einstein College of Medicine, Bronx, NY, USA

## Abstract

**Background:**

The number of students selecting careers in primary care has declined by 41% in the last decade, resulting in anticipated shortages.

**Methods:**

First-year medical students interested in primary care were paired with primary care mentors. Mentors were trained, and mentors and students participated in focus groups at the end of each academic year. Quantitative and qualitative results are presented.

**Results:**

Students who remained in the mentoring program matched to primary care programs at 87.5% in the first year and 78.9% in the second year, compared to overall discipline-specific match rates of 55.8% and 35.9% respectively. Students reported a better understanding of primary care and appreciated a relationship with a mentor.

**Conclusions:**

A longitudinal mentoring program can effectively support student interest in primary care if it focuses on the needs of the students and is supportive of the mentors.

## Background

The anticipated shortage of physicians in the U.S. is expected to be particularly acute in the primary care disciplines [1,2]. Over the past decade,the number of medical students selecting careers in primary care has declined by 41% [3]. Given the anticipated shortage [4] primary care specialties have sought to understand this decline and promote student selection of their fields [5].

Many factors impact the career choices of medical students, yet effective means of influencing these choices have not been determined [6]. Hauer and colleagues identified characteristics related to the institution, the individuals, and their experiences in medical school that contributed to primary care career intent among more than 1100 senior medical students at various institutions [7]. In this sample, 23% of students planned careers in Internal Medicine (IM), yet only 2% selected primary care IM. Senf and colleagues [8] assessed students' career interest at matriculation and noted that ambulatory exposure was an important element influencing career choice for Family Medicine (FM). Using logistic regressions, Kassebaum and colleagues [9] examined data from the AAMC graduating student questionnaire and documented demographic factors, student attitudes, and institutional characteristics predictive of interest in a career in FM but were unable to identify predictors for general IM or general pediatrics (PED). They found no correlation with students' incurred debt level, institutional mission related to primary care, early clinical experience, or admission criteria considering primary care preference. They concluded that primary care career intention is largely determined by student interest and exposure to FM and ambulatory care.

Another factor that potentially influences career choice is student perception of clinician expertise. Campos-Outcalt highlighted the importance of student perceptions of faculty competence in FM [10,11]. Bland et al [12] in their meta-analysis of the specialty selection literature determined that most students enter medical school with an interest in primary care but that interest wanes as students are exposed mainly to specialist physician faculty. Exposure to ambulatory care has an impact on career choice, perhaps because it allows students to see primary care physicians demonstrating their particular area of expertise.

Mentoring has an important place in the preparation of medical students [13], and the impact of mentoring on students' career selection has been highlighted [14,15].The strengths and weaknesses of mentoring programs have been identified [16,17]. The varied goals and roles of mentors have been described [18] as well as the responsibilities of both the faculty mentors and the students being mentored. [19] "Best-practices" have been identified for both mentors [17] and mentees [20] and activities that facilitate or impede mentoring have been described [21]. However, much of the mentoring literature has focused on medical careers in academic settings or research institutions; we were unable to identify a study that looked at the effect of a mentoring program focused specifically on assisting undergraduate medical students with career selection (career decision-making) prior to residency selection.

In response to this gap, an elective extra-curricular mentoring program was established at a private medical school in the northeastern U.S. where match rates into primary care were persistently low compared to the rest of the U.S. This grant supported "Generalist Career Program" (GCP) was longitudinal over the four years of medical school, extending one year post-funding as an independent program and was designed to determine if early contact with a mentor, specifically a primary care clinician, would influence medical student career choice.

## Methods

According to our Institutional Review Board, the data presented here is program evaluation, does not meet the federal definition of research, and was not subject to IRB review.

Setting: Grant-supported mentor-mentee program developed at an urban private medical school in the northeastern US.

Participants and Activities: This interdisciplinary program was designed to recruit first year medical students (MS1) beginning in September 2005 and was funded through 2008. The grant supported 4 core faculty, representing the primary care disciplines of FM, IM and PED, and a program coordinator. Mentors affiliated with the medical school through local academic hospitals and community-based practices were recruited from each of these disciplines. The program expected to recruit approximately 25 students each year of the 3-year grant.MS1 year students were recruited for the GCP via e-mail, flyer, and an introduction to the program presented during freshman student orientation.Students who expressed interest in primary care and being mentored were invited to an information session where the program was described in detail. Interested students who were willing to commit to the program completed a mentee database questionnaire, used for the purpose of matching them personally and professionally to mentors. Primary care faculty mentors were recruited via general e-mail invitation, the alumni office, and direct contact by the core faculty.The GCP goals and objectives were shared with potential mentors, and responsibilities were outlined. Faculty also completed a mentor database questionnaire used for matching them to mentees. The selected faculty mentors were required to attend an in-person faculty development session to increase both their understanding of and comfort with mentoring and to review the goals of the program. The matching of mentors with mentees was organized by the GCP coordinator and core faculty: accommodating the mentee's request (most commonly requesting a mentor by field of practice/gender/family), and based upon matching outside interests (most commonly hobbies and community involvements). Mentors and mentees were notified of their pairing, and the mentors were asked to initiate a first meeting as soon as possible with their mentees. Mentors were encouraged to schedule on-going meetings away from the medical school over a meal or social activity to ease encounters. The GCP activities included: mentor-mentee meetings (monthly during the MS1&2 years and quarterly during the clinical MS3 year); didactic conferences (monthly during MS1&2 years and quarterly during the MS3 year); enhanced community-based primary care research opportunities (summer between MS1&2 year); regional/national meeting attendance (any year) with a primary care mentor or designated faculty; and participation in program evaluation. Mentors received monthly email updates on student life and curriculum, which served as triggers for potential topics of conversations, and highlighted opportunities for support during stressful academic periods. Mentors received a small stipend ($30/month if meeting occurred) to compensate their time and defray the costs of any activities shared.

### Program evaluation

This project collected both qualitative and quantitative data for evaluation. All students, whether or not they remained in the program (completers and non-completers) were asked to respond to a post-residency match survey to assess the influence of the GCP program and the impact of a mentor on their choice of residency. In addition, the mentees and mentors attended separate face-to-face focus groups annually. Focus group probes were developed by the GCP core faculty and moderated by non-MD faculty to avoid any conflict of interest. The data was digitally recorded and transcribed. Focus group transcripts and written evaluations were analyzed for recurring themes. Two researchers who were not part of the program verified themes.

## Results

### Quantitative

See Table 1 for demographics on the mentors and mentees.

**Table 1:** Demographics of grant participants

	Gender				Ethnicities		
	**Female N (%)**	**Male N (%)**	**Asian N (%)**	**Black N (%)**	**Caucasian N (%)**	**Hispanic N (%)**	**Other N (%)**

Mentees	53 (64)	30 (36)	22 (26.5)	7 (8.4)	45 (54.2)	5 (6)	4 (4.8)

Mentors	42 (65)	23 (35)	8 (12.3)	7 (10.8)	42 (64.6)	5 (7.7)	3 (4.6)

In the first year (Y1) cohort, there were 29 students, 16 (55.2%) of whom completed 3 years in the program (completers). Fourteen of the 16 completers (87.5%) matched in primary care areas. Of the 13 students (44.8%) who did not complete the program (non-completers), 7 (53.8%) chose primary care residencies. As a point of reference, for the entire medical school class of 2009, 82 of the remaining 147 students (55.8%) chose primary care residencies [Table 2].

**Table 2:** Student match choices

		Y1 2005	Y2 2006
Total students		N = 29 (100%)	N = 33 (100%)

GCP* Completers		16(55.2)	25 (75.7) **

	Match PC***	**14(87.5)**	**15/19 (78.9)**

	FM	2	3

	IM	8	4

	PED	4	7

	Match not PC (Specialties)	2	5

Non-Completers		13(44.8)	8 (24.2)****

	Match PC	**7(53.8)**	**3(37.5)**

	FM	0	0

	IM	5	2

	PED	2	1

	Match not PC (Specialties)	6	2

Class data minus those in GCP		147	145

	MatchPC	**82 (55.8)**	**52(35.9)**

* GCP = Generalist Career Pathway

** 6 students delayed graduation. Match rates calculated based on 25 students who entered the match in 2010.

*** PC = Residencies with Primary Care Potential (FM, IM, PED)

**** 3 students transferred, no match data available

In the second year (Y2) cohort, there were 33 students, of whom 25 (75.7%) completed 2 years in the program. 19 of these 25 completers actually selected residencies; the other 6 elected to delay graduation and their choices are not yet available. Fourteen of these 19 actual completers (73.6%) matched in primary care. There were 8 (24.2%) students in this cohort who did not complete the GCP program, 3 (37.5%) of whom chose primary care residencies. As a point of reference, for the entire medical school class of 2010, 52 of the remaining 145 students (35.9%) chose primary care residencies [Table 2]. At this time, data on residency selection is available only for Y1 and Y2 cohorts.

The selection of a residency with the potential to practice primary care for the prior 3 years at our institution were relatively consistent: Class of 2006, 46.1%; Class of 2007, 46.2%; Class of 2008, 41.9%: Class of 2009, 58.5%, and Class of 2010, 38.8%. For comparison, we provide data on two other similar private medical schools in our geographic region [Table 3].

**Table 3:** Historical data from comparable geographically similar medical schools

	2006	2007	2008	2009	2010
Study School	66/143 (46.1)	73/158 (46.2)	73/174(41.9)	103/176 (58.5)	69/178 (38.8)

School A	46/158 (29.1)	40/136 (29.4)	33/126 (26.2)	50/152 (32.9)	55/166 (33.1)

School B	47/135 (34.8)	45/126(35.7)	47/111 (42.3)	28/105 (26.6)	36/115 (31.3)

Matching in Primary Care/Total students in class (%)

Based on a post-match survey, 7 of the 16 program completers of the Y1 cohort (43.7%), and 18 of 25 of the Y2 cohort (72%), stated that the GCP/mentor influenced their career choice.Only 1 student among the non-completing group, from the Y2 class, reported a positive connection between the program and career choice. There were 4 non-responders to the survey, 1 among the completers and 3 among the non-completers.

### Qualitative

Both mentors and mentees provided qualitative feedback during separate face-to-face end-of-year focus group sessions.By the end of the 3-year grant period, a total of 9 focus groups were conducted for overall program evaluation.Over 3 years of annual focus groups, there were a total of 6 faculty and 6 student groups conducted, with 8-10 participants per group for program evaluation. Annual formative feedback from these sessions suggested a number of changes, which the GCP instituted each year. In addition, we developed and distributed an ongoing list of "Best Practices" for mentors and mentees, stimulating improvements in the program (see Additional file 1). Two main themes with several sub-themes that emerged from the transcripts are described below.

### Theme #1: Impact on the understanding of primary care

Through the GCP experience, students were able to gain a better understanding of what the delivery of primary care requires and the responsibilities of being a primary care provider.

*Relationship with patients*: Students were able to think more about the relationships a primary care provider (PCP) develops with his/her patients as illustrated by a student who commented:

"I think it's ... the continuity that you have with your patient, that you actually form a relationship with them and therefore you are maybe more able to influence them and also manage their care better because you know their whole story."

*Comparison to specialty areas*: Students were able to reflect on the similarities and differences between primary care and other specialties. One student stated that primary care is also a "specialty".

"Different than other specialties, just like each specialty is different. But primary care is a specialty in my opinion. And the scope of knowledge is quite great which is scary to many people I think. ....because people want to be experts ... it's just the way our society is. .... And you may not be an expert, but if you know how to ask questions and find out information you're really quite skilled. I think primary care teaches you that. Because you can't know everything possible."

*Challenges of primary care in medically underserved urban communities*: Students learned how issues such as lack of health insurance, time constraints, language barriers, and shortages of physicians limit patients' access to health care and increase the burden on primary care providers. One student summed up some of the frustrations that PCPs face:

"Practicing in underserved communities...makes the primary care physician's job so much harder to manage ....if you .....get a patient who has so many problems because they've never had good health care until the time that they're 50 or 60. ...they have so many problems that could have been prevented and it's extremely inefficient."

*Significance of primary care: *Students developed a deeper appreciation for the services primary care providers offer patients. According to a second year student:

" ... even the most minimal care that you give is probably more than a lot of these patients could receive elsewhere."

### Theme #2: Facilitators and inhibitors of the mentoring relationship

Several factors influenced whether or not the mentors and mentees were able to build a positive relationship.

*Opportunity to view mentors in various roles*: Students found it useful to view mentors' different roles in various settings. This allowed students to understand that choosing primary care does not limit them from exploring other opportunities.

"I got to see her in different roles. I got to see her when she was seeing patients, and then another time I just happened to go, and it was more like residents were reporting to her, and so I saw her in that role, and then we had time to talk in between that, and then she had to go to [see]- - the neonates so then I got to see her in that. It was nice to see the various roles she had as a pediatrician."

*Not just about medicine*: Students valued the conversations with their mentors on aspects of their lives besides medicine. Students were able to know their mentors not just as health care providers, but also as individuals.

"And I found that just talking to someone in the profession and getting to see what ...aspects [they enjoy] or what they don't enjoy, or how .. they have time to fit this in or how they have time to fit that in or how did they get to the decision to go into primary care, that kind of stuff. And it's been good."

The focus group data also addressed the process of initiating and maintaining a longitudinal mentor-mentee relationship. There were, however, some challenges identified that the program needed to address:

*Mismatch of expectations and responsibilities*: There were differences between the mentor and mentee in terms of the kinds of mentorship activities expected and how the relationship should be managed.

"My mentor wanted me to meet her while she was practicing. I wanted a mentor to talk to and have someone answer question[s]. I did not want to shadow."

*Lack of time*: Due to competing demands for both mentors and mentees, scheduling meetings was a major obstacle in developing and sustaining the relationship.

"And it is sometimes a little hard to get in touch with him but when we go out we have a really great time..... I wish we could get together more, but we are both really busy so it is difficult."

## Discussion

Many students enter medical school with a professed interest in a career in primary care. While it is anticipated that students reassess residency and career choices during medical school, many factors work to derail their initial interest: future remuneration, perception of a controllable lifestyle, exposure to specialist faculty, and a hidden agenda suggesting the best and brightest should pursue subspecialty training [7-9].The GCP program was designed to determine if early contact with a mentor, specifically a primary care clinician, would influence medical student career choice toward primary care by nurturing and supporting their interest over the span of their medical education.

Our student data support a positive impact of a longitudinal mentoring program, beginning in MS 1 year and continuing through graduation, influencing student interest in primary care careers. Our data showed on average that one third more students, starting with an initial interest in primary care, actually entered primary care fields when they sustained a mentoring relationship with a primary care physician. The students had a better idea of what being a primary care practitioner entailed, had an increased respect for the field, and recognized the impact of health disparities on patients and their physicians.

One of the strengths of our program was its interdisciplinary focus: family medicine, pediatrics and internal medicine were all represented in the core leadership for the program and among the mentors.In addition, the conceptual framework of the mentorship program included matching mentors and mentees based on their personal backgrounds and self-identified interests. Starting the relationship from entry into medical school and the continuing support over three, and sometimes four, years of school was challenging but we believe contributed to the positive outcomes. The individualized mentoring relationship, allowing for consideration of both the personal and professional roles of clinicians, was a reported strength. Many mentors selected were teaching faculty, accomplished and respected by peers and patients, and the pride they took in their primary care practices was recognized by the students.

Establishing communication channels between individual mentor-mentee pairs that worked effectively and efficiently, and supported meetings, was challenging and required direction from the program coordinator. Mentors were typically not directly connected to the timeline of mentees' medical school experiences. We had hoped that a mentor-mentee list-serve would support a virtual communication space, but it was never used by students or faculty. Focused prompts to mentors to assure they were informed of the mentees "life cycle" in medical school was identified as a critical component, so we switched to monthly email alerts that focused on the mentee's current curriculum and activities. This effective communication channel between all key players (program coordinators, mentees and mentors) was established early and maintained to assure desired outcomes. Mentors reported these prompts as key to helping initiate a connection to their mentee and providing "talking points" to begin a conversation that could then continue to supportive dialogues and relationship building. Lastly, early identification and alignment of both mentor and mentee expectations was key to program success, specifically in maintaining longitudinal relationships that shift in purpose over time and can adjust easily to student development. This was evident in the data generated from both mentor and mentee focus groups and supported annual program improvements.

Other studies of mentorship during medical school have not addressed impact on specialty career selection. It is difficult to determine the impact of any discrete intervention. Data from the AAMC Graduating Students Questionnaire for 2008 [22] and AMCAS match results since 2009 [23], indicate that the number of students selecting residencies in primary care specialties has increased slightly for the past two years, making it even more difficult to determine if the GCP program was responsible for the changes in match rates we observed.

One of the limitations of our study is the inability to accurately predict primary care career selection based on medical school match rates.Not all students who enter a residency program from which they could practice primary care will do so. Currently, approximately 20 to 25 percent of internal medicine residents eventually choose to specialize in general internal medicine, compared with 54 percent in 1998.Among pediatric residents completing training, 40 percent planned to pursue a career in primary care according to 2008 data, with similar findings among senior residents scheduled to complete training in 2009 [24].We would need to follow our mentees through their residency training period to determine how many actually practice in a primary care setting, and to accurately identify how the mentoring program affected their career decisions. However, we do know that 2 of 14 (14.3%) completers in Y1 and 3 of 19 (15.8%) in Y2, chose family medicine and thus will likely remain in primary care. No non-completers went into family medicine, and from the rest of the class for each of those years 2/147 or 1.4% in the Y1 and 3/145 or 2.1% in the Y2 years selected family medicine. These numbers are very small, and only suggest further study is necessary. In theory, we may have skewed the impact of the mentoring program by merely identifying those students entering school who were thinking about primary care early in their medical school career and funneling them into the mentoring program. There were, however, students interested in primary care upon entrance to medical school and not interested in being mentored, and students who sometime during their medical school years elected primary care as a career choice without direct mentorship. However, those students who did not complete the mentorship program made similar choices to the rest of their class for the match, with approximately half choosing primary care; while 85.7% of those remaining in the mentorship program chose primary care. Indeed, we had 2 students who completed the program, one each year, matched to OB-Gyn with the intent to use this pathway to the primary care of women. The perception that Ob-Gyn as a PC specialty vs. a surgical subspecialty needs to be considered as a confounding variable in future research on career choice. We are aware that students might have declared interest in primary care to obtain a mentor. This is unlikely to have happened often: students were required to attend monthly didactic sessions that were primary care oriented as well as meet with their mentor.

In spite of our best efforts to have students and faculty notify us of their meetings, it did not happen often enough that any estimate could be made of frequency: no "dose response" could be determined. Matching both medical and outside interests created strong connections, and eachdyad developed the encounters they most valued.

Of interest is that both among our mentees and mentors, 2/3 were women. We have no way of knowing the cause, but we might hypothesize this is due to the fact that women are more relationship oriented, more willing to ask for help, more concerned with balancing home and work, and/or tend to work on a very practical level. There is significant literature on gender differences in mentoring [25,26] and women's ways of knowing [27]. Many of our female mentees requested a female mentor. It would be interesting to include gender differences in longitudinal mentoring in future studies.

Our program and outcomes focused on the early initiation and longitudinal aspect of mentoring relationships for medical students. The program leadership recognized the changes inherent to more knowledge and clinical exposure over four years of medical school, with student needs of the mentor changing in kind. Mentoring on these changing levels was consistently reinforced with the mentors to foster a positive outcome. The students' selection of a residency in a primary care discipline was an important outcome for both the program and the individual mentors who invested their time and nurtured their relationships with their mentees.More important was the recognition by program leadership that mentoring is a valuable component to medical student education.

## Conclusion

A mentoring program geared to the support of medical students going on to residency training in primary care can be fulfilling to the faculty and students if a structured but flexible environment is established that focuses on the needs of the mentees and is supportive of the mentors. The mentoring program may have had significant impact in sustaining student interest in pursuing a primary care career, at least in the initial selection of a residency training program. Hopefully, our outcomes and Best Practices Tip Sheets (Additional file 1) for mentors and mentees will guide other schools establishing mentoring programs and ease the "start-up" pains for both students and faculty.

## Competing interests

The authors declare that they have no competing interests.

## Authors' contributions

DD is the primary investigator on the grant and designed the study. Authors DD, DI, LR, MS contributed equally to the project: solicited/matched mentors and mentees; created/supervised mentor-mentee programming; assessed data and drafted manuscript. AF contributed to study design and created the evaluation; provided mentor training; ran the focus groups for mentors and mentees; contributed to the manuscript

WHL assisted with the qualitative data analysis and contributed to the manuscript. All authors read and approved the final manuscript.

## Pre-publication history

The pre-publication history for this paper can be accessed here:

http://www.biomedcentral.com/1472-6920/11/27/prepub

## Supplementary Material

Additional file 1 Appendix 1. Click here for file
