# Supplementary material for: The influence of longitudinal mentoring on medical student selection of primary care residencies
Source: BMC Med Educ. 2011 Jun 2;11:27. doi: 10.1186/1472-6920-11-27 (PMC3128853; doi:10.1186/1472-6920-11-27)
Supplement: Additional file 1 — Appendix 1. [file 1472-6920-11-27-S1.DOC]

**Appendix 1**

**A good mentor**

1. Develops a comfortable relationship with mentee facilitating the asking of both professional and personal questions; is open and informal
2. Explains the diversity of a career in medicine (settings, disciplines, roles)
3. Consults with mentee as a resource for achieving personal and professional goals
4. Is available to discuss non-medical aspects of being a doctor and what different health professions career choices mean; tolerates ambiguity and uncertainty in terms of student career interest; can refer to other disciplines in medicine for advice
5. Is available to mentee-answers emails in a realistic time frame and is flexible as possible in scheduling meetings; uses email when meetings cannot be scheduled to keep in contact
6. Accepts the role of “aggressor” with student- i.e. initiates repeated contact (important due to perceived power differential by mentee)
7. Is able to offer mentee the opportunity to shadow mentor in professional role
8. Provides longitudinal relationship to mentee
9. Knows mentee well enough to write a strong letter of recommendation, based on both a personal and professional relationship.

**A good mentee**

Is prepared for a meeting with mentor based on mutual agenda (work, social) of mentor and mentee

1. Dresses professionally for meetings
2. Negotiates with mentor best format for ongoing communication (email, text, social networking sites)
3. Takes initiative to come to meetings prepared and on time
4. Shares common interest or extracurricular interests with mentors

**Key mentoring tasks**

1. Listen and help student reflect
2. Be a resource based on needs of mentee
3. Provide emotional support during periods of academic stress
4. Be a sounding board for mentee ideas/thoughts and support mentee’s development
5. Give career guidance
6. Assist with summer/elective planning to enhance exploration of medicine
7. Initiate life experience discussions as well as pointed academic guidance
8. Proof read essays for applications, if asked
9. Write letter of recommendation, if asked
